# Development of ELISA Using Phage-Displayed Stx2 Mini-Body for Detection of STEC Antigen in Field Farming Pig Samples

**DOI:** 10.3390/microorganisms13020382

**Published:** 2025-02-09

**Authors:** Jin Hur, Ho-Kyoung Jung, Jung-Ho Park, Anoth Maharjan, Seung-Won Park

**Affiliations:** 1 Department of Veterinary Public Health, College of Veterinary Medicine, Jeonbuk National University, Iksan-si 54596, Jeollabuk-do, Republic of Korea; hurjin@jbnu.ac.kr; 2 CTCVAC Inc., 106, Saengmyeonggwahakgwan-gil, Hongcheon-eup, Hongcheon-gun 25142, Gangwon-do, Republic of Korea; pignvet@naver.com; 3 Bio-Evaluation Center, Korea Research Institute of Bioscience and Biotechnology, Cheongju-si 28116, Chungcheongbuk-do, Republic of Korea; jungho@kribb.re.kr (J.-H.P.); kretrem8722@nate.com (A.M.); 4 Department of Biomedical Science, Daegu Catholic University, Gyeongsan-si 38430, Gyeongsangbuk-do, Republic of Korea

**Keywords:** sandwich ELISA, Stx2e mini-body, STEC antigen, edema disease

## Abstract

Porcine edema disease (ED), which causes enormous economic losses in pig farms, is caused by Shiga toxin type 2e (Stx2e) *Escherichia coli* (STEC), which frequently occurs in young piglets. In this study, we aimed to express a fused Stx2e peptide on a phage surface to generate an innovative sandwich ELISA for the detection of STEC antigen in field pig farming samples. The amino acid sequences at positions 241–319 were selected for capture antibody (T1D2) production. T1D2 was selected after the third round of biopanning, and it showed a high yield with no major impurities. T1D2-ELISA can detect recombinant modified Stx2e antigen, and the detection limit of the antigen was approximately below 20 pg/mL. The sensitivity of T1D2-ELISA was determined using five different stool samples, with a total of 25 stool samples. Positive Stx2e antigen samples were detected only in one of the 25 samples using T1D2-ELISA. The ELISA values of positive stool samples were >300 pg and <600 pg. In conclusion, we developed an innovative ELISA for the detection of STEC antigens in field pig farming samples. It can also be used to easily detect STEC antigens in porcine stool samples. We anticipate that our novel T1D2-ELISA method will enable the effective monitoring of STEC antigen content during industrial vaccine production. By leveraging this approach, we aimed to enhance production efficiency and ensure high-quality vaccines.

## 1. Introduction

Shiga toxins (Stxs) are AB_5_ toxins composed of a pentamer B subunit non-covalently linked to a mono-enzymatically active A subunit. It is known to bind to a specific receptor on the cell surface, called glycosphingolipid globotriaosylceramide [1,2]. Ten subtypes of Shiga toxins (Stx1a, Stx1c, Stx1d, and Stx2a-Stx2g) exist as two immunologically different heterogeneous types, Stx1 and Stx2, sharing the same structure and function with approximately 50% homology; however, neither is cross-neutralized by antibodies [1]. Stx2 types include Stx2a (formerly named Stx2), Stx2c, Stx2d, and Stx2e [1,3,4]. Stx2e is mostly associated with porcine edema disease (ED), whereas others are associated with a higher risk of human diseases [1,5,6].

In pigs, Shiga toxin type 2e (Stx2e), produced by *Escherichia coli* (STEC), is responsible for both diarrhea and edema. This toxin plays a crucial role in gastrointestinal symptoms observed in affected animals [7]. STEC is derived from *E. coli* strains that have acquired the ability to produce Shiga toxin through gene transfer by Shiga-toxin (Stx) phages [1]. Pigs infected with Shiga toxin-producing STEC, particularly those exposed to elevated levels of Stx2e toxin, may manifest swelling in different anatomical regions, including the digestive tract, eyelids, and brain [8]. Evidence of vascular endothelial damage caused by this swelling includes motor weakness, lying down, seizures, and paralysis [8,9]. This ultimately leads to a high mortality rate in pigs infected with Stx2e-producing *E. coli* [8,9,10,11]. Hence, the observation that ED is the most pathogenic condition among porcine *E. coli* infections underscores the importance of STEC as a major foodborne pathogen. Additionally, the risk of serious zoonotic infections with pigs as vectors highlights the need to thoroughly investigate the presence and characteristics of these pathogenic isolates in pig populations [7,12,13].

Several monoclonal and polyclonal type-specific antibodies have been developed against the unique antigenicities of Stx1 and Stx2 [14,15]. Some of these antibodies have been characterized in terms of their binding specificity and reactivity to two types of antigens [14,15,16,17]. Currently, commercially developed Stx antibody-based immunoassays for STEC detection cannot detect all subtypes of Stxs (Stx2c, Stx2e, Stx2f, and Stx2g) produced by different STEC serotypes [14,18]. A recently developed enzyme-linked immunosorbent assay (ELISA) kit for STEC detection can distinguish between Stx1 and Stx2 toxins, and ELISA kits using specific monoclonal antibodies can detect all subtypes [14].

Phage display technology, first developed in 1985, involves the introduction of a gene that codes for the fusion of a desired protein peptide into the phage coat genes [19,20,21,22,23,24,25]. In this technique, the target protein is present on the phage [21]. This phage display technology has been reported in several studies, and peptides or proteins can be used as antigens in antibody detection tests [19,21,22,23,24,25]. ELISA is the most common method for detecting infectious antigens or antibodies following an immune response [19,26]. This method uses stable reagents and inexpensive equipment; therefore, its sensitivity, specificity, and reproducibility are high, and the results are highly accurate [19,20,26]. Phage display technology is widely used to produce highly specific monoclonal antibodies. The advantage of this technology is that antibodies can be produced in a relatively short time without the need for animals [27,28]. Based on the antigen-binding site, antibody fragments such as antigen-binding fragments and single-chain variable fragments were constructed in a phage display library format [27,29]. Phage-displayed antibodies have higher resistance to harsh environmental conditions compared to antibodies produced by other methods, and they maintain uniformity for a longer period compared to recombinant proteins expressed in bacterial hosts [21,30]. Their small size facilitates the large-scale production of antibodies in bacterial expression systems [31]. Taken together, these results indicate that phage display technology may be an important alternative for producing diagnostic antibodies [27].

In this study, we aimed to express a Stx2e peptide fused to the phage surface and then use this recombinant phage to develop an innovative ELISA diagnostic method. We expect that the newly developed T1D2-ELISA method can effectively monitor the STEC antigen content during industrial vaccine production, thereby increasing the efficiency of high-quality vaccine production. Additionally, this method can be used to detect STEC antigens in field samples from pig farms.

## 2. Materials and Methods

### 2.1. Experimental Samples

In a previous study, we expressed and purified recombinant mStx2e protein, which was used as a standard protein for T1D2-ELISA [26,31]. Briefly, the mStx2e antigen contains the Stx2eA-fragment, and Stx2eB proteins were co-expressed in *E. coli* BL21 (DE3) pLysS-competent cells. The purified target protein was dialyzed using dialysis buffer (20 mM Tris-HCl, 200 mM NaCl, and 5% glycerol, pH 7.4), concentrated via centrifugation using an Amicon Ultra Scientific Filter (Merck, Taufkirchen, Germany), and subjected to protein quantification (Bradford protein).

Five to six fecal samples were collected from each breeding section of a pig farm comprising 120 sows in Gimcheon, Gyeongsangbuk-do, Republic of Korea. Fecal samples were collected with priority given to diarrheal feces; however, if there was no diarrhea, normal feces were collected depending on the breeding section. Additionally, four nursing piglet fecal samples with positive reactions were requested by the Manufacture Facility and R&D Center CTCVAC Co., Ltd. (Hongcheon-gun, Republic of Korea) for producing an autologous *E. coli* vaccine for diarrhea in nursing piglets at a pig farm with 300 sows in Pocheon, Gyeonggi-do, Republic of Korea.

### 2.2. Antibody Production Using the Phage Display Technique

Antibody production services were obtained from YntoAb, Inc. (Seongnam-si, Gyeonggi-do, Republic of Korea). During biopanning, the chicken library was aligned using purified protein. The antigen was used after synthesizing two parts of Stx2e with the peptide and conjugating them with BSA. The target protein (antigen) was immobilized onto a solid surface followed by the incubation of phage from the antibody library with the immobilized target protein. After incubation, the unbound phages were washed off and high-affinity phages were eluted from the immobilized antigen. The eluted phages were then incubated with *E. coli* host cells to allow for infection and amplification of a subpopulation of phage-displaying antibodies with affinity for the target. All experiments were repeated thrice. Colonies of the infected bacteria were grown on plates that produced monoclonal scFv from each colony. Once the scFv monoclonal was produced, the binders were selected using an ELISA based on STEC.

The scFv-fragment-positive binders were amplified using PCR and then transferred into the mini-body (scFv-Fc) expression vector. The resulting recombinant plasmids were transfected into 293F cells, and monoclonal Stx2e mini-body antibodies were purified using protein G affinity chromatography. After dialysis with PBS, the antibody concentration was measured using a NanoDrop 2000 spectrophotometer (Thermo Fisher Scientific, Waltham, MA, USA). The purified mini-body antibody (T1D2) was analyzed using 0.1% SDS-12.0% gradient PAGE under reducing conditions.

### 2.3. T1D2 Specificity Test Using Sandwich ELISA

A 96-well plate was coated with the T1D2 mini-body (2 μg/mL) at 37 °C for 1 h and then blocked with 3.0% BSA. Each mStx2e antigen (1 μg/mL) was added into microplates coated with the optimal mini-body antibody. Shiga-like toxin 2 antibody (Biorbyt, Waltham, UK) as the first detection antibody was diluted at 1:1000 in reagent diluent, and 100 μL of reagent diluent was found using optical concentration of phage particle. Next, 100 μL per well of goat anti-rabbit IgG-Biotin conjugate (1:5000, Thermo Fisher Scientific) was added. Following this, the samples were detected according to the ELISA optimization conditions established through preliminary experiments. A standard curve between the optical density (OD) at 450–630 value and the concentration of Stx2e protein standard was developed, and the detection limit was confirmed.

### 2.4. Determination of the Optimal Condition for Sandwich ELISA Using Absolute Quantitative Analyze

In sandwich ELISA, recombinant mStx2e proteins were used as antigens [26,31]. To evaluate the sensitivity of the ELISA in detecting STEC antigen, 9 times in a 2-fold series starting from an initial concentration of 600 pg/mL in PBS (0.01 M, pH 7.2) was performed. In total, 100 µL of each dilution of 600, 300, 150, 75, 37.5, 18.75, 9.375, 4.6875, 2.34375, and 0 pg/mL recombinant mStx2e protein standard was added to the microplate coated with the optimal mini-body antibody. Subsequent experiments were performed as described above (Section 2.3). The experiment was repeated in triplicate.

### 2.5. Evaluation for the Specificity of Sandwich ELISA

For the diagnostic specificity test, 25 stool samples collected from CTCVAC, Inc. (Hongcheon-gun, Gangwon-do, Republic of Korea) were evaluated. Samples from five different types of uncharacterized, clinically look-alike pigs, reproductive female pigs (*n* = 5), nursing piglets (*n* = 5), weaning pigs (*n* = 5), porkers (*n* = 6), and random province pigs (*n* = 4), were included to evaluate the diagnostic specificity. A sandwich ELISA was optimized to detect antigens against STEC antibodies in porcine samples. Clear polystyrene 96-well microplates (R&D Systems, Minneapolis, MN, USA) were coated with the Stx2e mini-body antibody (1:1000) as the capture antibody at an original concentration of 824 µg/mL using a coating buffer (NaCl 8 g/L, KCl 0.2 g/L, Na_2_PO_4_ 1.15 g/L, pH7.2–7.4, 0.2 µm filtered) and incubated at 4 °C overnight. After washing three times with a washing buffer consisting of PBS (pH7.2) and 0.05% Tween-20, the plates were blocked with reagent diluent (1.0% BSA in PBS pH7.2–7.4, 0.2 μm filtered; ThermoFisher Scientific) and incubated at room temperature for 1 h and then washed again three times with washing buffer. Stool samples were diluted at 1:10 in reagent diluent, and 100 μL of reagent diluent was added to the plates. After incubation in a dark chamber at room temperature for 2 h, the plates were washed three times with the washing buffer. Shiga-like toxin 2 antibodies (Biorbyt) as the second antibody was diluted at 1:1000 in reagent diluent, and 100 μL of reagent diluent was added to the plates and 100 μL per well of goat anti-rabbit IgG-Biotin conjugate (ThermoFisher Scientific) diluted to 1:5000 was added. The plates were incubated at room temperature for 2 h and washed three times, and 100 μL per well color reagent A (H_2_O_2_) and color reagent B (Tetramethylbenzidine) (ThermoFisher Scientific) as substrate solutions (ThermoFisher Scientific) were used for detection. The plates were incubated for 20 min, 50 μL per well of stop solution (2N H_2_SO_4_) was added to stop the reaction, and absorbance was read at 450 nm (BioTek microplate reader, Agilent Ltd., Santa Clara, CA, USA).

## 3. Results

### 3.1. Peptide Amino Acid Sequences and Purification of Anti-Stx2 Mini-Body

**Figure 1:**
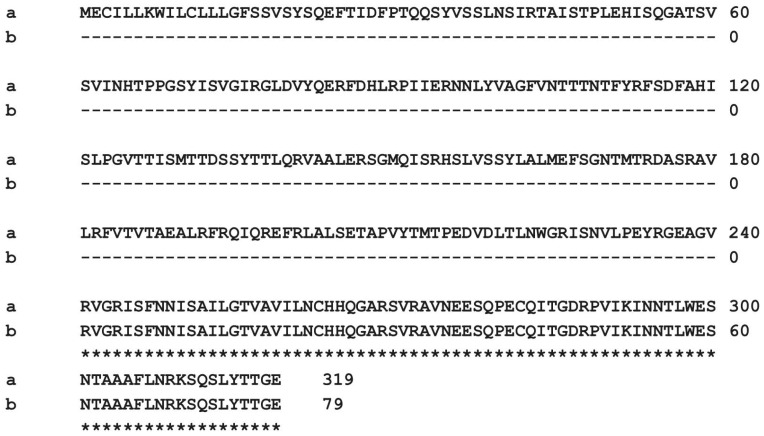
Multiple alignment of the C-terminal parts of proteins that share sequence similarity with the C-terminal part of T1D2. a. Full-length amino acid sequences, b. mini-body T1D2 amino acid sequences.

*E. coli* Shiga-like toxin 2e variant A and B subunit genes (NCBI accession number U72191.1) were chosen as target antibody proteins for this study. The selected amino acid sequence alignments at positions 241–319 are shown in Figure 1.

**Figure 2:**
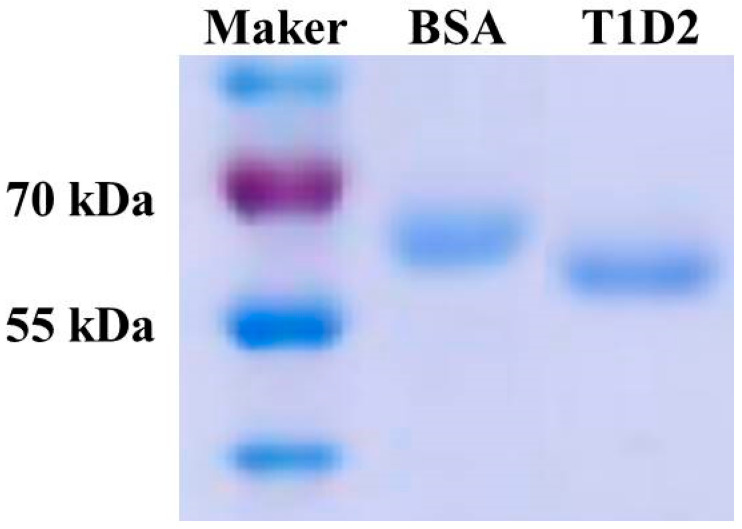
SDS-PAGE analysis of the purified mini-body T1D2. The mini-body T1D2 was expressed and purified using a column with protein G resin. A clear T1D2 band appeared on 12.0% SDS-PAGE using Coomassie blue staining.

To isolate a specific antibody against Stx2e protein, a human mini-body antibody phage library was used. The library of V_H_ and V_L_ displayed on M13 phages was screened through biopanning, followed by evaluation using ELISA. The results of enriched Stx2e-specific phages showed that the maximum enrichment occurred after the third round compared to the values for control panning. Following the screening process, 90 of the 96 positive colonies were identified for specific binding to the Stx2e protein using ELISA. The Stx2e mini-body antibody (T1D2) was expressed and purified using a protein G resin column. Coomassie blue staining using 12.0% SDS-PAGE revealed clear bands (Figure 2). After purification, the yield of purified mini-body T1D2 could be up to 824 μg/mL. As shown in Figure 2, SDS-PAGE revealed no major impurities.

### 3.2. Purified T1D2 Specificity Test

Initial studies focused on the specificity of the target mini-body T1D2 for ELISA detection limits. First, polystyrene 96-well plates were used to pre-coat T1D2 (2 μg/mL). The wells were designated T1D2 coating and uncoating wells, respectively. mStx2e antigen (1 μg/mL) was diluted with reagent diluent, and each 100 μL of reagent diluent with or without T1D2 was added to each of the two wells. As shown in Table 1, the mStx2e antigen was detected only in T1D2-coated wells. These data indicated that sandwich ELISA using T1D2 is specific for Stx2e detection.

### 3.3. Determination of the Optimal Condition for Sandwich ELISA Using Absolute Quantitative Analyzer

**Figure 3:**
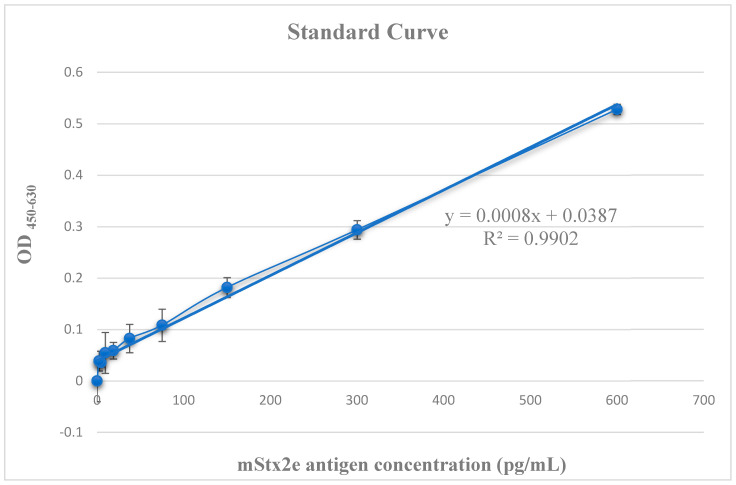
Standard curve of T1D2-ELISA for absolute quantitative analysis. The standard curve was assayed using two-fold serially diluted mStx2e antigen with concentrations of 600, 300, 150, 75, 37.5, 18.75, 9.375, 4.6875, 2.34375, and 0 pg/mL. ELISA standard curve of optical density against mStx2e antigen concentration with absorbance was measured at 450 nm with a reference wavelength of 630 nm. The concentration (pg/mL) is shown on a logarithmic scale. The coefficient of determination (R^2^) was 0.9902. The experiment was repeated in triplicate on two separate occasions.

The standard curve of ELISA using T1D2 (T1D2-ELISA) was analyzed using two-fold serially diluted mStx2e antigens at concentrations of 600, 300, 150, 75, 37.5, 18.75, and 0 pg/mL. The standard curve between the O.D_450–630_ value and concentration of the antigen protein was obtained as follows: Y = 1025X − 30.705, R^2^ = 0.9836. The lowest detectable concentration for antigen diagnosis was approximately 20 pg/mL. The standard curve is shown in Figure 3.

### 3.4. Field Test for Collected Farming Pig Samples Using Sandwich ELISA

To evaluate the specificity of T1D2-ELISA, Stx2e was detected in stool samples using T1D2-ELISA. We collected 5 different and a total of 25 stool samples from CTCVAC Inc., Republic of Korea. Five different types of porcine stool samples (sows, nursing piglets, weaned pigs, finishing pigs, and nursing piglets producing an autologous *E. coli* vaccine) with uncharacterized clinical features were included to evaluate the diagnostic specificity (Table 2). The results in Table 2 showed that the O.D. values of all stool samples against Stx2e were negative, except for the mStx2e antigen-positive random province sample, suggesting that T1D2-ELISA is specific to antigens against Stx2x. The sensitivity of T1D2-ELISA was determined using stool samples. The limit of detection of T1D2-ELISA was diluted 1:10 in stool samples. This finding indicates that the T1D2-ELISA is an easily useful and applicable tool for stool samples.

Additionally, to measure the amount of antigens in random stool samples, T1D2-ELISA was performed by comparing stool samples diluted to 1:10 with recombinant mStx2e antigens at two different concentrations (300 and 600 pg). As shown in Table 3, the O.D. values of the random province stool samples were >300 and <600 when compared with those of the recombinant antigen at two different concentrations.

## 4. Discussion

The primary considerations in developing Stxs detection systems are high accuracy, reliability, and short analysis time [32]. Additionally, the developed system should be usable quickly and accurately by personnel with minimal training [32]. The current methods for detecting STEC are broadly categorized into approaches based on microbiology, nucleic acid sequencing, immunology, and biosensing systems [32]. Microbiological culture methods are commonly used in clinical samples to efficiently detect STEC. The advantages of these methods include relatively low cost and simplicity. However, they are time-consuming, taking more than four days to obtain results due to multiple steps such as pre-enrichment, selective enrichment, selective and differential plating, serological confirmation, and biochemical screening [32,33]. New gene amplification methods such as PCR, quantitative real-time PCR (qPCR), and loop-mediated isothermal amplification (LAMP) are known for their rapid and highly specific detection of *Stx* genes using primers specific to *stx1* and *stx2* genes. These methods are also cost-effective [32,34,35,36,37,38,39]. However, DNA-based assays cannot detect the expression levels of Stx or differentiate between active and inactive toxins, and they have not yet received Food and Drug Administration (FDA) approval for diagnosing human STEC infections in clinical laboratories [32,40].

Enzyme immunoassays (EIA), first introduced in the United States in 1995, have been primarily used in clinical laboratories to detect STEC [32]. The main advantages of EIA are the ability to identify all serotypes and provide faster results than culture-based methods by detecting Stx directly from samples or enrichment cultures [32]. To date, six FDA-approved EIAs are available for detecting Stx in human specimens [32]. Among them, two commercial detection kits, Premier EHEC (Meridian Diagnostics Inc., Colorado Springs, CO, USA) and ProSpecT Shiga Toxins Microplate Assay (Remel Inc., Lenexa, KS, USA), are based on Sandwich ELISA [32]. Both kits can detect Stx1c, Stx1d, Stx2c, and Stx2f variants produced by STEC but cannot detect Stx2d and Stx2e variants [32]. Additionally, a validated method for a sandwich ELISA system using anti-Stx antibodies has been developed and used to diagnose all Stx subtypes produced by STEC in samples such as processed meat [32,41].

In the present study, *E. coli* Shiga-like toxin 2e variant A subunit and B subunit genes were chosen as target antibody proteins for amino acid sequences at positions 241 to 319 (Figure 1). The selected amino acid was the Stx2eA2 region, which is very important in the formation of the Shiga toxin structure, where the pentamer formed from five Stx2eBs is connected to one Stx2eA2 [26,42]. Studies have reported that immunization with the Stx2e B subunit protein as a vaccine antigen to block toxin binding induces antibodies against the B subunit, but the B subunit alone induces almost no neutralizing antibodies against Stx2e compared to the holotoxin [42,43]. Additionally, serum obtained from immunization with the Stx2 B subunit monomer was reported to be unable to recognize Stx2 [42,44]. These reports and data suggest that the multimeric structure of the B subunit is important for maintaining neutralizing epitopes. Therefore, the pentameric structure of the B subunit is considered crucial for efficiently inducing neutralizing antibodies against Stx2e [42].

Enrichment of Stx2e-specific phages was observed after the third round of biopanning. T1D2 cells were selected from 90/96 positive colonies after expression and purification. T1D2 showed a high yield and no major impurities in SDS-PAGE analysis (Figure 2). The current study was conducted to evaluate a novel T1D2-ELISA using T1D2 as a capture antibody against the Stx2e porcine antigen. The preliminary results demonstrated that T1D2-ELISA could detect the recombinant mStx2e antigen (Table 1). It also showed that T1D2 contained enough antibodies for the detection of T1D2-ELISA. T1D2-ELISA was performed using a two-fold serially diluted mStx2e antigen to generate a standard curve (Figure 3). The antigen detection limit was <20 pg/mL (Figure 3). To evaluate the specificity of T1D2-ELISA, we collected five different and 25 stool samples. Only one Stx2e antigen-positive sample was detected by T1D2-ELISA. The sensitivity of T1D2-ELISA was determined using stool samples to determine the limit of detection of T1D2-ELISA. To measure the amount of antigen in the positive stool sample, T1D2-ELISA was performed by comparing stool samples diluted to 1:10 with recombinant antigens at two different concentrations (300 and 600 pg). The ELISA value of the positive stool samples was higher than 300 pg and lower than 600 pg when compared with that of the recombinant antigen at two different concentrations (Table 3). With the advancement of genetic engineering technology, we can produce recombinant antibodies. Mini-bodies are genetically engineered antibody fragments composed of two single-chain antibodies (scFv) connected by the human IgG1 CH3 domain (single-chain Fv-CH3 dimer, ~80 kDa) [45]. This phenomenon can be realized by fusion with the CH3 domain through the immunoglobulin hinge region, allowing the formation of disulfide-bonded covalent dimers and providing antigen-binding domains that significantly increase binding affinity [45]. Compared to full-length antibodies, mini-bodies offer several advantages, including smaller molecular weight, better penetration, faster clearance, and higher targeting [45,46]. Mini-bodies can be utilized in a wide range of applications in biomedicine, particularly in tumor diagnosis and therapy [45,47,48]. In this study, we produced the T1D2 mini-body antibody using genetic recombination methods. Unlike traditional methods, T1D2 can be mass-produced from microorganisms because it does not require animal cells for antibody production. This is crucial as a strategy to increase cost-effectiveness in the development of the T1D2-ELISA system.

In conclusion, we expressed the Stx2e peptide fused to a phage surface and used this recombinant phage to develop an innovative sandwich ELISA method for the effective detection of antigens against STEC in field swine farm samples or vaccine production sites. We expect that using this method, we will be able to diagnose STEC antigens on pig farms, monitor the content of STEC antigens during vaccine production, and improve production efficiency and vaccine quality. In addition, the production of antibodies has transitioned to recombinant antibodies, and the methods used in this study, such as phage display, are evolving to include AI-based epitope prediction. To respond to new variants and use the results as training data for machine learning, we aim to use the findings of this study as the basic structure to produce new detection antibodies.

## Figures and Tables

**Table 1:** The specificity test of target mini-body T1D2 for sandwich ELISA system.

T1D2 (Mini-Body)	O.D_450–630_			
	mStx2e Antigen (1 µg/mL)		Background Control	
2 µg/mL	3.059	3.008	0.047	0.048

**Table 2:** The specificity evaluation of Stx2e Sandwich ELISA system using pig farm stool samples.

Samples Information		Number of Total Samples	T1D2 ELISA Data	
			Number of Negative Samples	Number of Positive Samples
Sows	Pig farm in Gimcheon, Gyeongsangbuk-do	5	5	-
Nursing Piglets		5	5	-
Weaned Pigs		5	5	-
Finishing Pigs		6	6	-
Nursing Piglets with producing an autologous *E. coli* vaccine	Pig farm in Pocheon, Gyeonggi-do	4	3	1

**Table 3:** Measurement of the antigen amount in positive sample.

Samples	Dilution Factor	O.D_450–630_	Amount of Protein (pg)
Positive Stool Sample	1:10	0.085	560
mStx2e Antigen		0.056	300
		0.123	600

## Data Availability

The original contributions presented in this study are included in the article. Further inquiries can be directed to the corresponding author.
